# Unbiased metabolome screen leads to personalized medicine strategy for amyotrophic lateral sclerosis

**DOI:** 10.1093/braincomms/fcac069

**Published:** 2022-03-17

**Authors:** Sarah Boddy, Mahjabin Islam, Tobias Moll, Julian Kurz, David Burrows, Alexander McGown, Anushka Bhargava, Thomas H Julian, Calum Harvey, Jack NG Marshall, Benjamin PC Hall, Scott P Allen, Kevin P Kenna, Eleanor Sanderson, Sai Zhang, Tennore Ramesh, Michael P Snyder, Pamela J Shaw, Christopher McDermott, Johnathan Cooper-Knock

**Affiliations:** 1 Sheffield Institute for Translational Neuroscience (SITraN), University of Sheffield, Sheffield, UK; 2 Department of Neurology, Brain Center Rudolf Magnus, University Medical Center Utrecht, Utrecht, The Netherlands; 3 Medical Research Council (MRC) Integrative Epidemiology Unit (IEU), University of Bristol, Bristol, UK; 4 Center for Genomics and Personalized Medicine, Stanford University School of Medicine, Stanford, CA, USA

**Keywords:** Metabolome, amyotrophic lateral sclerosis, Mendelian randomization, isoleucine, vitamin B12

## Abstract

Amyotrophic lateral sclerosis is a rapidly progressive neurodegenerative disease that affects 1/350 individuals in the United Kingdom. The cause of amyotrophic lateral sclerosis is unknown in the majority of cases. Two-sample Mendelian randomization enables causal inference between an exposure, such as the serum concentration of a specific metabolite, and disease risk. We obtained genome-wide association study summary statistics for serum concentrations of 566 metabolites which were population matched with a genome-wide association study of amyotrophic lateral sclerosis. For each metabolite, we performed Mendelian randomization using an inverse variance weighted estimate for significance testing. After stringent Bonferroni multiple testing correction, our unbiased screen revealed three metabolites that were significantly linked to the risk of amyotrophic lateral sclerosis: Estrone-3-sulphate and bradykinin were protective, which is consistent with literature describing a male preponderance of amyotrophic lateral sclerosis and a preventive effect of angiotensin-converting enzyme inhibitors which inhibit the breakdown of bradykinin. Serum isoleucine was positively associated with amyotrophic lateral sclerosis risk. All three metabolites were supported by robust Mendelian randomization measures and sensitivity analyses; estrone-3-sulphate and isoleucine were confirmed in a validation amyotrophic lateral sclerosis genome-wide association study. Estrone-3-sulphate is metabolized to the more active estradiol by the enzyme 17β-hydroxysteroid dehydrogenase 1; further, Mendelian randomization demonstrated a protective effect of estradiol and rare variant analysis showed that missense variants within *HSD17B1*, the gene encoding 17β-hydroxysteroid dehydrogenase 1, modify risk for amyotrophic lateral sclerosis. Finally, in a zebrafish model of *C9ORF72*-amyotrophic lateral sclerosis, we present evidence that estradiol is neuroprotective. Isoleucine is metabolized via methylmalonyl-CoA mutase encoded by the gene *MMUT* in a reaction that consumes vitamin B12. Multivariable Mendelian randomization revealed that the toxic effect of isoleucine is dependent on the depletion of vitamin B12; consistent with this, rare variants which reduce the function of *MMUT* are protective against amyotrophic lateral sclerosis. We propose that amyotrophic lateral sclerosis patients and family members with high serum isoleucine levels should be offered supplementation with vitamin B12.

## Teaser

Boddy et al. report an unbiased screen of the metabolome using Mendelian randomization to identify underlying determinants of risk for amyotrophic lateral sclerosis. Three metabolites are significant after multiple testing correction: oestrone-3-sulphate and bradykinin were protective and isoleucine was harmful. Estrone-3-sulphate and isoleucine were significant in follow-up analyses.

## Graphical Abstract

**Graphical abstract:**
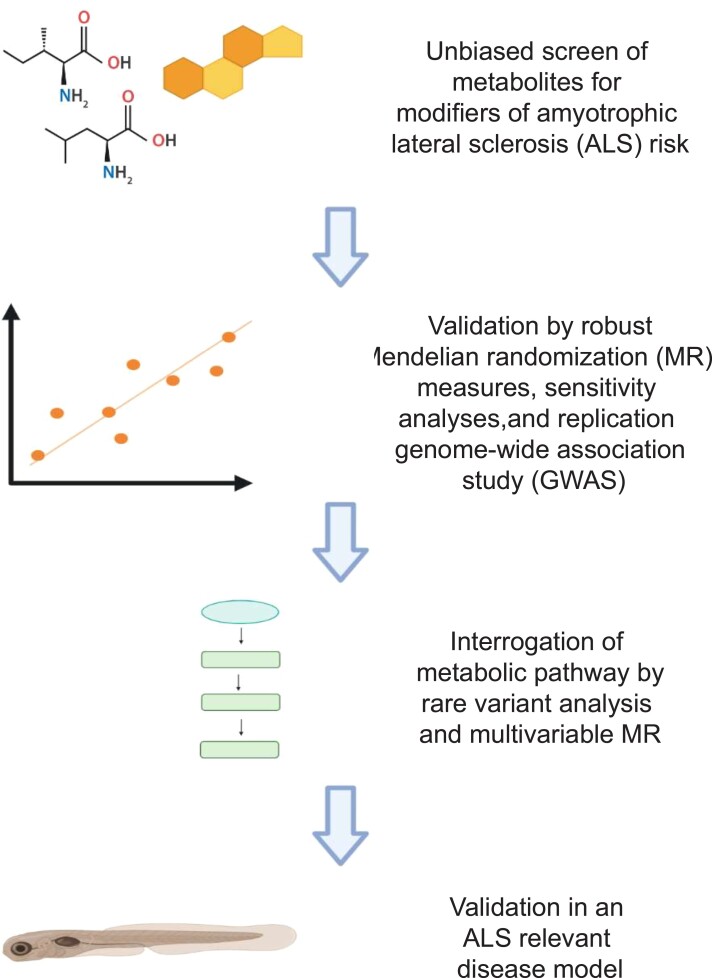

## Introduction

Amyotrophic lateral sclerosis (ALS) is an incurable and rapidly progressive neurodegenerative disease that affects 1/350 individuals in the UK. The cause of ALS is unknown but the majority of cases are thought to result from a complex gene–environment interaction, likely as a series of sequential ‘hits’.^1^ Much progress has been made to describe the genetic basis of ALS^2^ but few environmental risk factors have been conclusively demonstrated. To understand the interaction between genetics and the environment, each can be profiled in turn but this leads to an exponential increase in the number of possible combinations and an often intractable multiple testing problem. An alternative is to seek biological readouts which integrate genetic and environmental influences, such as the metabolome, transcriptome and microbiome.^2^

Metabolites that make up the metabolome are the intermediates and end products of cellular regulatory processes. Changes in the levels of metabolites are influenced by both genetic background and environmental stimuli.^3^ For example, the serum concentration of a given metabolite may be lower because of genetic mutations within enzymes responsible for key steps in metabolite synthesis, or because of a limited supply of substrates from the diet. ALS has been associated with a number of metabolic defects including deficits in the production of nicotinamide^4^ and inosine.^5^ Indeed metabolites such as branched-chain amino acids (BCAA)^6,7^ and vitamin B12^8^ have been administered to ALS patients as part of experimental medicine trials.

A key question to address in the study of the metabolome is to differentiate upstream causes of neurodegeneration from downstream consequences. The metabolome is not stable over time and therefore cross-sectional studies struggle to determine which changes are truly causal. Two-sample Mendelian randomization (MR) has been used extensively to infer causal relationships between exposures, such as serum metabolite concentrations, and disease risk including ALS.^9–11^ MR can address causality because each exposure is measured by genetic instruments which are fixed at conception, and are therefore necessarily upstream of a late age of onset disease such as ALS. Ultimately MR is a test for a dose–response relationship between genetic liability to a particular exposure and genetic liability to an outcome such as ALS; this measurement is used to infer or refute a causal relationship.

**Figure 1:**
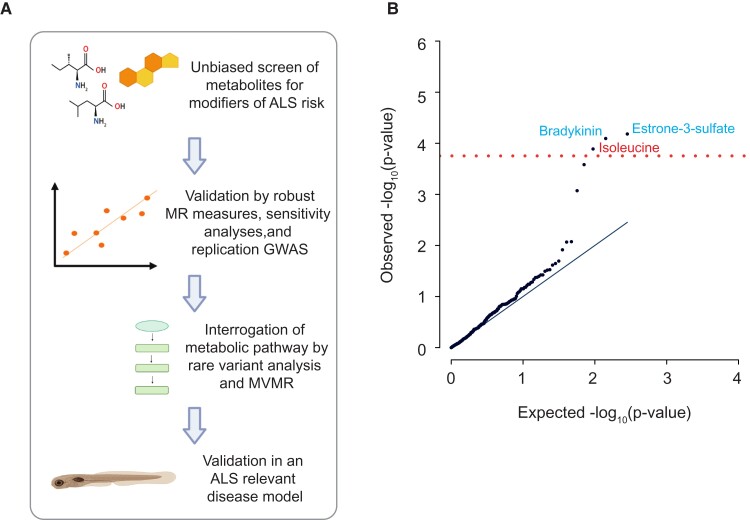
**Unbiased screen for metabolites linked to ALS risk.** (**A**) Schematic of the study design: We performed an unbiased screen of 566 metabolites using Mendelian randomization (MR) to infer a causal relationship with ALS. Metabolites that passed stringent multiple testing correction were further evaluated using robust MR measures, sensitivity analyses and a validation GWAS. Next, for metabolites that passed all measures, we evaluated other members of their metabolic pathway using orthogonal techniques which utilize common and rare genetic variants so as to infer the underlying biological mechanism. Finally, we evaluated proposed mechanisms of neurotoxicity/neuroprotection in an ALS-relevant disease model. MVMR refers to multivariable Mendelian randomization in which multiple exposures are considered simultaneously. (**B**) QQ-plot demonstrates that there was no evidence of *P*-value inflation in our unbiased screen (*λ* = 1.11); three metabolites were significant after Bonferroni’s multiple testing correction (red line). Blue text denotes a protective association, whereas red text denotes a harmful association.

A rapid rise in the availability of genetic instruments to measure both metabolites^12,13^ and ALS^14,15^ led us to perform an unbiased screen of the metabolome using MR. In this context, ‘unbiased’ refers to the hypothesis-free testing of a complete set of metabolites rather than a hypothesis-based approach focusing on candidate metabolite(s). After stringent multiple testing correction, we identified three metabolites with a significant effect on ALS risk: estrone-3-sulphate, bradykinin and isoleucine. These changes are consistent with existing literature. We were particularly interested in isoleucine because of the potential for therapeutic manipulation. We used multivariable MR to demonstrate that the toxic effect of isoleucine is dependent on the depletion of vitamin B12. We have supported our data using a complementary genetics approach in which we identified rare missense mutations associated with ALS which alter the function of enzymes that metabolize estrone-3-sulphate and isoleucine either directly or indirectly. Finally, we present evidence that estradiol, a more active metabolite of estrone-3-sulphate, is neuroprotective in a zebrafish model of ALS. Our approach is summarized in Fig. 1A. Our work has the potential to lead to safe and cost-effective personalized medicine: ALS patients and family members with high serum isoleucine levels may benefit from vitamin B12 supplementation.

## Materials and methods

### Two-sample Mendelian randomization

Genetic instruments used to measure lifetime serum metabolite levels were derived from publicly available GWAS.^12,13^ Unbiased screening utilized an ALS GWAS consisting of 12 577 ALS patients and 23 475 controls.^14^ This is the largest published ALS GWAS to date where a linear mixed model (LMM) was used to correct for population variation;^16^ it has been previously demonstrated that an LMM can achieve improved power over a meta-analysis without significant false positives.^14^ Replication was performed using a larger but more heterogeneous ALS meta-analysis GWAS of 20 806 ALS patients and 59 804 controls.^15^

Genetic instruments used to measure exposures are chosen based on an arbitrary *P*-value cut-off.^17,18^ A cut-off that is too low will lose informative instruments, but a cut-off that is too high could introduce non-informative instruments and is more likely to lead to instrument pleiotropy. We choose to use a relatively liberal (*P* < 5e−06) *P*-value threshold to select instrumental SNPs so as to maximize power to detect significant associations. We have previously shown that a conservative threshold can lead to an underpowered test and paradoxical results.^9,19^ In the current work, the risk of invalid SNPs was mitigated by robust MR measures and sensitivity analyses as described below. Identified SNPs within a 10 kb window were clumped for independence using a stringent cut-off of *R*^2^≤0.001 within a European reference panel; where SNPs were in linkage disequilibrium (LD), those with the lowest *P*-value were retained. Where an exposure SNP was unavailable in the outcome dataset, a proxy with a high degree of LD (*R^2^* ≥ 0.9) was identified in LDLink within a European reference population.^20^ The effects of SNPs on outcomes and exposures were harmonized in order to ensure that the beta values were signed with respect to the same alleles. For palindromic alleles, those with minor allele frequency (MAF) > 0.42 were omitted from the analysis in order to reduce the risk of errors due to strand issues.^21^

In our unbiased metabolome screen, we reported the IVW (multiplicative random effects) estimate of causal inference for all MR tests because this carries the most statistical power. To ensure that we did not include false-positive results in our unbiased screen we measured the inflation factor (*λ*) which is the ratio of the observed median *P*-value to the expected median *P*-value. Under the assumption that the majority of statistical tests will be non-significant then *λ* should ∼1. It is expected that the majority of metabolites are unrelated to ALS risk and therefore this is a reasonable assumption. Before calibration, there was evidence of significant *P*-value inflation (*λ* = 1.38, Supplementary Fig. 1A). The optimal number of instrumental SNPs was tuned to minimize *λ*; this process excluded analyses that used *n* < 6 or *n* > 14 SNPs (Supplementary Fig. 1B) and removed problematic *P*-value inflation (*λ* = 1.11, Fig. 1B). Tests including small numbers of SNPs give excessive weight to single SNPs, whereas tests including large numbers of SNPs are more likely to include heterogeneous or outlier SNPs to which the IVW estimate is particularly vulnerable.^22^ Alternatively, heterogeneous SNPs can be detected using sensitivity tests; we applied the Cochran’s Q-test and excluded all analyses where the Cochran’s Q-test *P*≦0.05 in addition to those with *n* < 6 instrumental SNPs. In this analysis, the genomic inflation was less effectively controlled (*λ* = 1.62); there was no change in the metabolites exceeding the Bonferroni threshold.

To increase confidence in the IVW results from our unbiased screen, we performed a series of robust MR measures and sensitivity analyses. The aim of this process was to detect evidence of instrument pleiotropy and/or heterogeneity which could invalidate the IVW effect estimates. MR measures such as the weighted median,^23^ weighted mode,^24^ MR Lasso^25^ and MR Egger^26^ are relatively robust to the presence of invalid SNPs. The MR-Egger intercept test determines whether there is directional horizontal pleiotropy. The MR-PRESSO global test determines if there are statistically significant outliers within the exposure-outcome analysis.^27^ The *I*^2^ is a measure of heterogeneity between variant-specific causal estimates, with a low *I*^2^ indicating that Egger is more likely to be biased towards the null.^28^ Finally, we performed a leave-one-out (LOO) analysis to determine if any single SNP(s) were exerting a disproportionate effect.^29^

**Figure 2:**
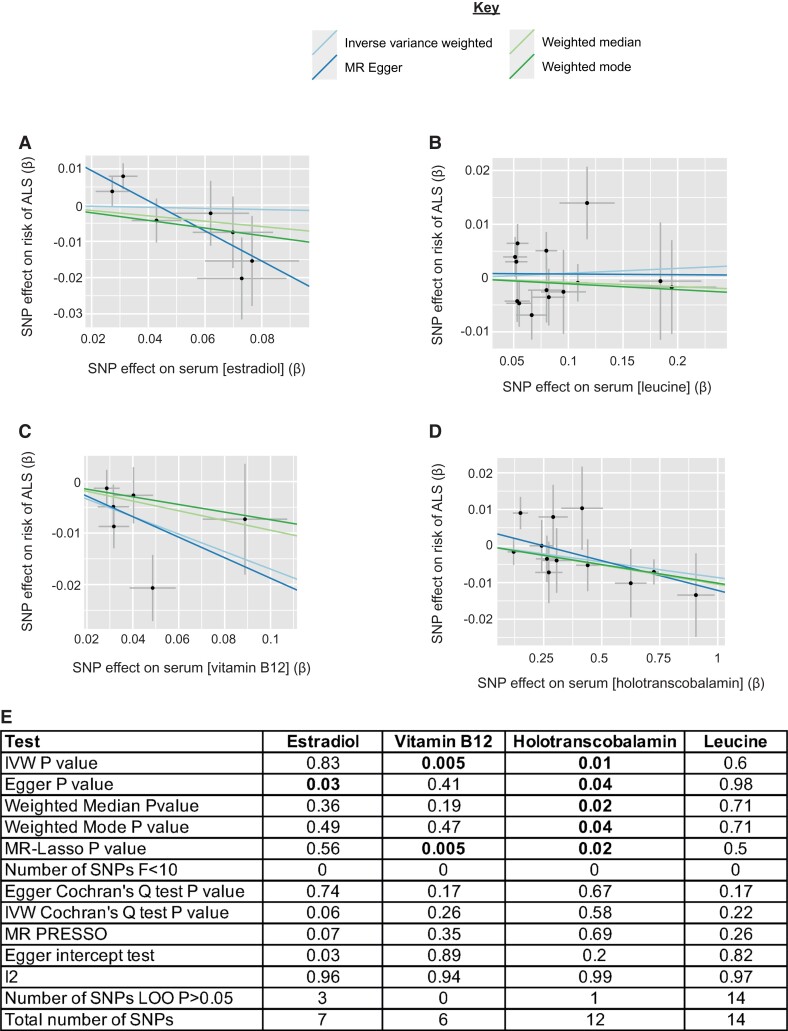
**MR analysis of additional members of metabolic pathways containing metabolites identified in an unbiased screen.** (**A–D**) Scatter plots demonstrating the correlation between genetic liability to serum estradiol (MR Egger *P* = 0.03, beta = −0.42, se = 0.14) (**A**), leucine (IVW *P* = 0.6, beta = 0.009, se = 0.017) (**B**), vitamin B12 (IVW *P* = 0.005, beta = −0.17, se = 0.06) (**C**), and holotranscobalamin (IVW *P* = 0.01, beta=-0.08, se = 0.004) (**D**), with genetic liability to ALS. Points indicate effect size (β) and standard errors for each SNP-outcome relationship. (**E**) Robust MR measures and sensitivity analyses for each MR analysis.

The MR Egger estimate was preferred to the IVW estimate for the MR analysis of estradiol because the IVW estimate is likely to be invalid as indicated by the Egger intercept (Egger intercept = 0.02, se = 0.006, *P* = 0.03, Fig. 2E). The MR Egger estimate indicated a significant relationship (MR Egger *P* = 0.03, beta = −0.42, se = 0.14, Fig. 2A and2E). However, a necessary condition for a valid MR Egger estimate is the fulfillment of the InSIDE (INstrument Strength Independent of Direct Effect) assumption^30^ which states that pleiotropic effects for instrumental SNPs are independent of association with the exposure of interest; in practice, it is difficult to be sure that this is the case^31^ and so an isolated MR Egger result should be interpreted with caution.

All MR analyses were carried out using the TwoSampleMR (version 0.5.6)^32^ and Mendelian Randomization (version 0.5.1)^33^ R (version 4.0.4) packages.

### Multivariable MR

MVMR^34,35^ was used to test whether the toxic effect of isoleucine was conditional on depletion of either vitamin B12 or its active form, holotranscobalamin. GWAS summary statistics were obtained for two-sample MR analyses. The *P*-value cut-offs used to choose instrumental SNPs for each exposure were chosen so as to achieve adequate instrument strength for both exposures (conditional *F*-statistic >10 for each exposure^36^). Reported results showed no evidence of instrument heterogeneity (Cochran’s Q-test *P* > 0.05). Exposures were derived from independent cohorts^13,37^ and therefore a correction for the covariance between the effect of the genetic variants on each exposure was not necessary. MVMR was implemented using the MVMR (version 0.3)^35^ and MendelianRandomization (version 0.5.1)^34^ R packages.

### Calculation of genetic covariance and linkage disequilibrium score regression

LDSC is used to evaluate the genetic correlation between traits using GWAS summary statistics. LDSC was implemented using the LDSC package (version 1.0.1).^38,39^ SNPs were filtered according to the presence in HapMap3 and MAF > 1%; strand ambiguous SNPs were removed.

### Identification of rare deleterious variants and burden testing

Rare variant burden testing was performed using the Project MinE Databrowser.^40^ Variants were determined to be rare if MAF < 1% in the ExAC population dataset.^41^ Association testing was performed using Firth logistic regression, including sex, sequencing platform and the first 10 principal components as covariates. For each gene, two different burden tests were performed: First we considered the total set of missense variants; and then we considered only ‘disruptive’ LOF variants including frame-shift, splice site, exon loss, stop gained, stoploss, startloss and transcription ablation variants as described in.^40^

### Treatment of C9ORF72 Zebrafish with estradiol

Adult and larvae zebrafish (*Danio rerio*) were maintained at 28.5°C and bred according to established procedures.^42^ Animal protocols were undertaken in line with a Home Office-approved project licence. The care and maintenance of animals were performed under the Home Office project licence as per the animals (scientific procedures) act of 1981 (ASPA). *C9ORF72* 2.2-7 zebrafish^43^ were crossed with AB non-transgenic zebrafish and selected for expression of the *C9ORF72* expansion prior to the experiment onset by GFP expression. At 2 days post-fertilization (dpf) embryos were treated with one of a positive control: riluzole (10 µM, *n* = 16) or inosine (30 mM, *n* = 24); a negative control (DMSO at 0.1%, *n* = 15); or estradiol (10 µM, *n* = 21) (All drugs from Sigma-Aldrich). A comparison was made with non-transgenic zebrafish embryos (*n* = 24). Riluzole concentration and sample sizes were derived from our previous work;^43^ the estradiol concentration was chosen to match the Riluzole concentration; inosine concentration was higher based on observed dose–response *in vitro.*^5^ Each embryo was kept individually in a 96-well plate (96 well, µClear, Grenier Bio-One) and chronically dosed by immersion in 200 µl volume of drug solution from 2dpf until 5 dpf at 28°C. At 5dpf embryos were transferred into ‘V-bottom’ 96-well plates (Greiner Bio-One) in 50 µl volume before sonication at 25% amplitude for 5 s (Vibracell, Sonics and materials). Plates were centrifuged (1300 RCF for 10 min; CWS ALC PK120 centrifuge, T536 rotor) and 20 µl of the supernatant was loaded onto 384-well plates (Grenier Bio-One) and measured on a Pherastar plate reader (BMG Labtech) for GFP (485 nm excitation/520 nm emission) and DsRed fluorescence (540 nm excitation/590 nm emission).

### Statistical analysis

Statistical tests applied to MR analyses are described in detail above. For the zebrafish ALS model DsRed expression, data are presented as mean with standard deviation from two independent clutches of fish; all data were tested for normality prior to Kruskal Wallis H-test with Dunn’s multiple testing correction.

### Data availability

All GWAS summary statistics were accessed through the IEU Open GWAS Project.^44^ GWAS identifiers for the metabolome screen are included in Supplementary Table 1. Other exposure identifiers used are: ‘ukb-d-30800_irnt’ for estradiol, ‘ukb-b-19524’ for vitamin B12, and ‘prot-a-2939’^37^ for holotranscobalamin. ALS GWAS identifiers are: ‘ieu-a-1085’^14^ and ‘ebi-a-GCST005647’.^15^

## Results

### Unbiased screen for metabolites with an effect on ALS risk

We obtained summary statistics from genome-wide association studies (GWAS) of serum concentrations of 566 metabolites^12,13^ which were population matched with a GWAS study of ALS.^14^ For each metabolite, we performed two-sample MR using a liberal threshold for instrument selection (*P* < 5e−06). Each instrument consists of a single nucleotide polymorphism (SNP) associated with the exposure of interest; signals from the group of SNPs are aggregated to provide an overall estimate of causality. We utilized an inverse variance weighted (IVW) multiplicative random effects estimate which offers a powerful and accurate causal inference under the assumption of limited balanced pleiotropy.^22^ Pleiotropy in this context refers to a causal relationship between an instrumental SNP and the outcome which is not mediated via the exposure of interest. Pleiotropy is detectable as heterogeneity between SNPs, in which the effect on the exposure is not proportional to the effect on the outcome. MR tests with very small or very large numbers of instrumental SNPs are more likely to contain heterogeneous or outlier SNPs which can produce false-positive IVW estimates.^23^ To avoid this problem, we excluded MR tests with small or large numbers of instrumental SNPs from our unbiased screen (**Materials and methods**). Before filtering there was evidence of significant *P*-value inflation (*λ* = 1.38, Supplementary Fig. 1A) but after filtering our results were not significantly inflated (*λ* = 1.11, Fig. 1B, Supplementary Fig. 1B).

The total set of results from our unbiased metabolome screen is presented in Supplementary Table 1. After Bonferroni multiple testing correction, three metabolites were significantly linked to ALS risk: Estrone-3-sulphate (IVW *P* = 6.58e−05, beta = −0.03, se = 0.008) and bradykinin (IVW *P* = 8.07e−05, beta = −0.05, se = 0.01) were protective; serum concentration of isoleucine was positively associated with ALS risk (IVW *P* = 1.29e−04, beta = 0.05, se = 0.01) (Fig. 1B). It is interesting that all of these results are consistent with the literature: ALS displays a male preponderance^45^ and estrone-3-sulphate is generally found at higher levels in females. Bradykinin has been suggested as the neuroprotective compound underlying ACE-I protection against ALS.^46^ Isoleucine is a BCAA which have collectively been associated with the acceleration of ALS progression.^6,7^

### ALS-associated metabolites pass sensitivity analyses and are significant in robust measures and a replication GWAS

**Figure 3:**
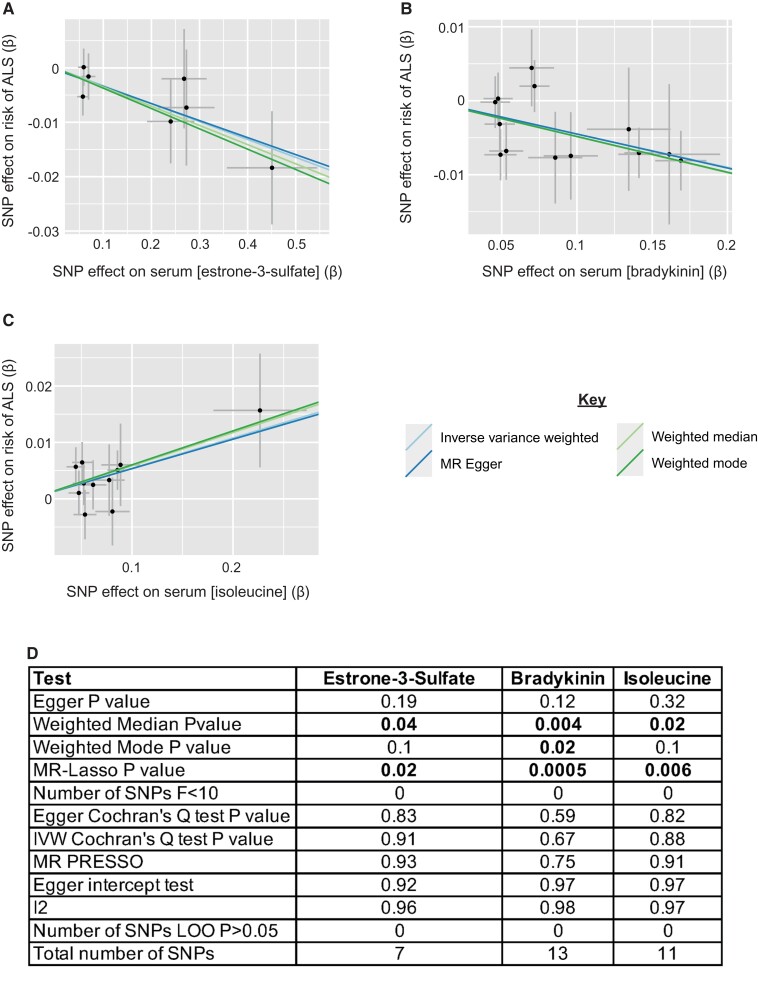
**Metabolites identified in an unbiased screen are significant in multiple robust MR measures.** (**A–C**) Scatter plots demonstrating the correlation between genetic liability to serum estrone-3-sulphate (IVW *P* = 6.58e−05, beta = −0.03, se = 0.008) (**A**), bradykinin (IVW *P* = 8.07e-05, beta = −0.05, se = 0.01) (**B**), and isoleucine (IVW *P* = 1.29e−04, beta = 0.05, se = 0.01) (**C**), with genetic liability to ALS. Points indicate effect size (β) and standard errors for each SNP-outcome relationship. (**D**) Robust MR measures and sensitivity analyses for each MR analysis.

The IVW measure for testing significance in MR is well powered but can be vulnerable to outlier SNPs.^23^ To support the reproducibility of identified ALS-associated metabolites, we applied a series of robust measures including weighted median,^23^ weighted mode,^24^ MR Lasso^25^ and MR Egger.^26^ All three exposures were significant in one or more robust measures (Fig. 3A–D, Supplementary Table 2). Moreover, sensitivity analyses confirmed that there was no significant instrument heterogeneity or pleiotropy and no outlier SNPs had undue influence (**Materials and methods**, Fig. 3D, Supplementary Table 2).

As an additional validation, we performed a phenotype-wide association study (PheWAS) of instrumental SNPs associated with estrone-3-sulphate, bradykinin and isoleucine. Searching for instrumental SNPs within the NHGRI GWAS catalog^47^ did not reveal any additional phenotype association for any instrumental SNP at the *P*-value threshold used for instrument selection in our unbiased screen (*P* < 5e−06). We also tested for association between instrumental SNPs associated with estrone-3-sulphate, bradykinin and isoleucine and any other metabolites included in our unbiased screen at the same significance threshold. For estrone-3-sulphate, no instrumental SNP was significantly associated with any other metabolite. For bradykinin, one instrumental SNP was associated with three other metabolites; repeating the MR analysis while excluding this SNP did not negate the identified association with ALS (IVW *P* = 2.0e−3, beta = −0.044, se = 0.014). For isoleucine, two instrumental SNPs were significantly associated with multiple other metabolites; repeating the MR analysis while excluding these SNPs did not negate the identified association with ALS (IVW *P* = 4.3e−03, beta = 0.053, se = 0.019).

Finally, as a further validation, we tested the three exposures in a different ALS GWAS;^15^ this GWAS is overlapping with the original GWAS used to identify ALS-associated metabolites but contains additional patients and controls. Estrone-3-sulphate (IVW *P* = 0.04, beta = −0.07, se = 0.03, Supplementary Fig. 2A and 2E, Supplementary Table 3) and isoleucine (IVW *P* = 0.001, beta = 0.12, se = 0.04, Supplementary Fig. 2C and 2E, Supplementary Table 3) were both significant in the second GWAS but bradykinin was not (IVW *P* = 0.3, beta = −0.06, se = 0.06, Supplementary Fig. 2B and 2E, Supplementary Table 3).

Overall robust measures and sensitivity measures, a PheWAS screen and analysis of a validation GWAS add confidence that discovered ALS-associated metabolites represent a true positive result.

### Estrone-3-sulphate is metabolized to estradiol which is associated with reduced ALS risk

Our unbiased metabolite screen revealed a protective effect of serum estrone-3-sulphate on the risk of ALS (Fig. 3A and3D, Supplementary Table 2). Estrone-3-sulphate is a naturally occurring estrogen that is converted continuously into estrone and estradiol,^48^ each of which has a higher binding affinity for estrogen receptors. However, estrone-3-sulphate is the most abundant circulating estrogen and it has a much longer half-life than its reactive products;^49^ therefore, it has been proposed that estrone-3-sulphate acts as a storage reservoir for these molecules.^50^ Estrone-3-sulphate makes up a large proportion of hormone replacement therapy (HRT) where it is used as a prodrug. We performed an MR in which we measured the effect of estradiol (**Materials and methods**), the most reactive estrogen, on the risk of ALS; higher serum levels of estradiol were protective against ALS (MR Egger *P* = 0.03, beta = −0.42, se = 0.14, Fig. 2A and2E) that suggests that estradiol may be the downstream effector molecule responsible for our observations regarding estrone-3-sulphate. In this case, the MR Egger estimate was preferred to the IVW estimate because the Egger intercept test (Egger intercept = 0.02, se = 0.006, *P* = 0.03, Fig. 2E) indicated that the IVW estimate is very likely to be biased.^31^ Moreover, the *I*^2^ is close to 1 (Fig. 2E) indicating that the MR Egger estimate did not suffer from weak instrument bias.^28^ In interpreting an isolated significant MR Egger estimate, some caution is necessary because it is not possible to be sure that the InSIDE assumption has been violated (**Materials and methods**). Estradiol has been previously shown to protect cultured motor neurons from excitotoxicity.^51^

### Estradiol supplementation leads to reduced cellular stress in an in vivo ALS model

**Figure 4:**
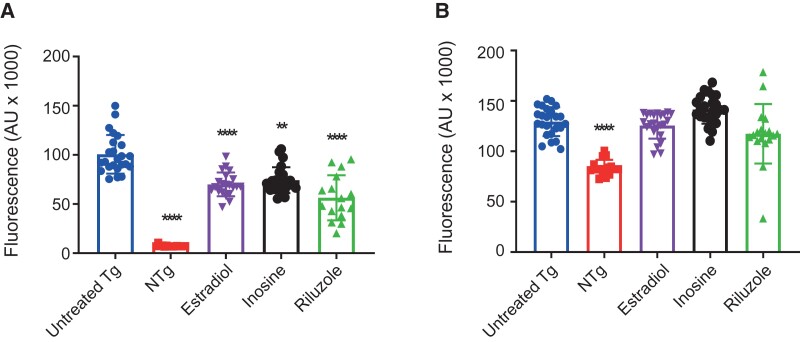
**Application of estradiol to a zebrafish model of *C9ORF72*-ALS reduces cellular stress.** (**A**) The effect of 3 days of metabolic supplementation on zebrafish DsRed expression with 10µM estradiol (30.4% reduction in DsRed expression, Kruskal Wallis H-test with Dunn’s multiple testing correction *P* = 0.0003), 30 mM inosine (26.1% reduction, *P* = 0.0024) or 10 µM riluzole (43.9% reduction, *P* < 0.0001). (**B**) The effect of metabolic supplementation on zebrafish GFP expression; no significant changes were observed. Untreated Tg indicates transgenic fish treated with 0.1% DMSO vehicle control. NTg indicates non-transgenic fish. Data presented as mean with standard deviation from two independent clutches of fish. ** *P*≤0.01. ****P≤0.001.

In an attempt to functionally validate our unbiased screen, we assessed whether estradiol supplementation was beneficial in an *in vivo* model of ALS. G4C2-repeat expansion of *C9ORF72* is the most frequent genetic variant of ALS.^52^ We have previously developed a zebrafish model of *C9ORF72*-ALS which recapitulates key molecular and behavioural phenotypes of ALS including motor decline and early mortality.^43^ Diseased fish carry a transgene to express the *C9ORF72* expansion fused inframe to GFP and in tandem, an *hsp70*-DsRed reporter for the activation of the heat shock cellular stress response (HSR). Activation of the HSR reporter is coincident with neurotoxicity, likely due to proteotoxic stress.^43,53,54^ We treated zebrafish embryos expressing a *C9ORF72* expansion with estradiol at day two post-fertilization (**Materials and methods**) and monitored cellular stress via the HSR reporter. Inosine^5^ and riluzole^55^ were included as positive controls. After 3 days of treatment, we observed a 30.4% reduction in DsRed expression (Kruskal Wallis H-test with Dunn’s multiple testing correction *P* = 0.0003, Fig. 4A) which was comparable to both inosine (26.1% reduction, *P* = 0.0024) and riluzole (43.9% reduction, *P* < 0.0001). We conclude that estradiol reduces proteotoxic stress within ALS neurons. No significant changes in GFP expression were observed with metabolic supplementation of estradiol, inosine or riluzole (Fig. 4B), indicating that supplementation had no effect on *C9ORF72* expression levels.

### Isoleucine toxicity is dependent on the depletion of vitamin B12

Our unbiased screen revealed a toxic effect of serum isoleucine (Fig. 3C and3D, Supplementary Table 2) whereby higher levels of isoleucine were associated with increased risk of ALS. In contrast, leucine, which is a structural isomer of isoleucine, is not associated with risk of ALS (IVW *P* = 0.6, beta = 0.009, se = 0.017, Fig. 2B and2E, Supplementary Table 4). Both leucine and isoleucine are BCAA and both are essential, i.e. they cannot be synthesized and must be obtained directly from the diet. Isoleucine and leucine have different downstream functions but both are ultimately metabolized to produce energy within the TCA cycle.^56^ We hypothesized that isoleucine toxicity may be a result of a downstream step in its metabolism which does not apply to leucine. Valine and isoleucine metabolism share certain similarities,^56^ however, MR of the effect of serum valine on ALS risk did not pass the quality control for our unbiased screen (**Materials and methods**). It is notable that isoleucine metabolism consumes vitamin B12, whereas metabolism of leucine does not.^57^ Indeed, the administration of isoleucine has been used as a clinical test to unmask borderline vitamin B12 deficiency.^58^ Vitamin B12 has been suggested as a treatment for ALS with some evidence of a positive effect on disease progression.^59^ As expected, MR for vitamin B12 revealed a protective effect on ALS (IVW *P* = 0.005, beta = −0.17, se = 0.06, Fig. 2C and2E, Supplementary Table 4, **Materials and methods**), and the effect was particularly marked for holotranscobalamin^37^ which is the active form of vitamin B12^60^ (IVW *P* = 0.01, beta = −0.08, se = 0.004, Fig. 2D–E, Supplementary Table 4, **Materials and methods**). The MR test of holotranscobalamin was significant in all robust measures (Fig. 2E, Supplementary Table 4) and in the replication ALS GWAS (Supplementary Fig. 2D–E).

We hypothesized that the effect of isoleucine on the risk of ALS might be mediated via depletion of vitamin B12. To test this hypothesis, we performed multivariable MR (MVMR)^34,35^ in which we included isoleucine and holotranscobalamin as exposures with ALS as the outcome of interest (**Materials and methods**). MVMR is a means of conditioning between alternative exposures in an MR study to determine which exposure is more closely linked to the disease outcome. Where two or more exposures are correlated this can be a powerful technique to elucidate underlying biological mechanisms. The toxic effect of isoleucine was non-significant when conditioned on levels of holotranscobalamin (isoleucine *P* = 0.14, beta = 0.04, se = 0.03 and holotranscobalamin *P* = 0.03, beta = −0.009, se = 0.004). The MVMR analysis achieved adequate instrument strength for both exposures and there was no evidence of instrument heterogeneity (**Materials and methods**). We used LD-score regression (LDSC) to show that there is negligible genetic correlation between serum isoleucine and holotranscobalamin (**Materials and methods**, genetic covariance = −0.001, se = 0.004) which supports the validity of our MVMR analysis. MVMR analysis of serum isoleucine with vitamin B12 gave the same result (isoleucine *P* = 0.07, beta = 0.06, se = 0.03 and vitamin B12 *P* = 0.02, beta = −0.16, se = 0.06). Our MVMR analysis supports the hypothesis that the toxic effect of isoleucine is conditional on the depletion of vitamin B12. We applied two different MVMR methods^34,35^ (**Materials and methods**) and both produced equivalent results.

### Rare missense variants in enzymes responsible for metabolism of estrone-3-sulphate and isoleucine modify risk of ALS

MR analysis relies on genetic instruments formed from common genetic variants which are usually not directly linked to protein function. We hypothesized that genetic mutations alter the function of enzymes responsible for the metabolism of estrone-3-sulphate and isoleucine, namely 17β-Hydroxysteroid dehydrogenase 1 (17β-HSD1) and methylmalonyl-CoA mutase, might have a significant impact on ALS risk if they interfere with downstream toxic or protective compounds. Mutations that directly alter protein function are typically rare^61^ and therefore we set out to discover rare ALS-associated genetic variants as a means of further interrogating the mechanisms linking estrone-3-sulphate and isoleucine to neurotoxicity.

To achieve adequate statistical power in our rare variant analysis, we aggregated variants into rare variant burden tests.^62^ We studied whole-genome sequencing (WGS) data from 4366 ALS patients and 1832 age and sex-matched controls.^40,63^ Rare variants were identified by population MAF <1%.^41^ 17β-HSD1 is a dimaeric enzyme responsible for the conversion of estrone-3-sulphate into estradiol; 17β-HSD1 is encoded by the gene *HSD17B1*. We discovered that missense mutations within *HSD17B1* were negatively associated with ALS risk (**Materials and methods**, Firth logistic regression *P* = 0.01, beta = −0.64, se = 0.25). We identified 29 missense variants (Supplementary Table 5) but none of these were classified as ‘disruptive’ loss of function (LOF) variants (**Materials and methods**).

*MMUT* encodes methylmalonyl-CoA mutase which catalyzes the isomerization of methylmalonyl-CoA to succinyl-CoA; methylmalonyl-CoA is a product of isoleucine but not leucine metabolism. Methylmalonyl-CoA mutase consumes vitamin B12 as a cofactor.^57^ Forty-eight missense rare variants were identified within *MMUT* of which 25 were ‘disruptive’ LOF mutations (**Materials and methods**, Supplementary Table 6). Both missense (*P* = 0.03, beta = −0.3, se = 0.2) and ‘disruptive’ LOF mutations (*P* = 0.02, beta = −0.7, se = 0.3) were significantly protective against ALS. Reduced function of methylmalonyl-CoA mutase might be expected to reduce consumption of vitamin B12 in response to isoleucine intake. The observation that reduced function of methylmalonyl-CoA mutase is protective against ALS is consistent with our conclusion that isoleucine-dependent vitamin B12 consumption is important in the development of motor neuron toxicity.

## Discussion

There have been a number of attempted unbiased profiles of the metabolome in ALS^64–68^ but typically these have not overcome multiple testing limitations and have therefore focused on broad pathways rather than specific metabolites. To our knowledge, no risk factor has been demonstrated conclusively using this approach. We have used MR to perform an unbiased screen of serum metabolites associated with the risk of ALS. MR gains power from large sample sizes and effective avoidance of problematic ascertainment bias. Our unbiased screen reveals three exposures which are significantly linked to the risk of ALS after stringent multiple testing correction: serum estrone-3-sulphate and bradykinin are protective but serum isoleucine is harmful. Each of these exposures is consistent with some previous literature, but each provides new insight into specific mechanisms and potential therapeutic targets. Our method provides good evidence for causality, but we note that MR is not usually a good tool for estimating effect sizes including a binary outcome measure such as ALS.^69^

Our unbiased MR screen utilized a relatively liberal threshold (*P* < 5e-06) for instrument selection. This was a specific choice in order to avoid false negatives due to inadequate instrument power. We have previously shown that a conservative threshold can lead to an underpowered test and paradoxical results.^9,19^ A liberal threshold is more likely to lead to instrument pleiotropy. To counter this, we used robust MR tests and sensitivity analyses to show that the ALS associations we have identified are not confounded by instrument heterogeneity. Specifically testing for pleiotropic SNPs by PheWAS analysis and then removing instrumental SNPs with multiple phenotype associations did not alter our key results despite a loss of power; indeed, our PheWAS screen demonstrated only limited overlap between genetic instruments associated with different metabolites at the *P* < 5e-06 threshold.

Males are at higher risk of ALS than females.^45^ Amongst females, an epidemiological study of 209 ALS patients and 672 controls demonstrated a reduced risk of ALS associated with a longer duration of exposure to endogenous estrogen.^70^ This is consistent with our finding that serum estrone-3-sulphate, the most abundant estrogen is protective against ALS. Estrone-3-sulphate acts as a reservoir for conversion to estradiol, a more active metabolite.^48,50^ Studies *in vitro* suggest that estradiol can prevent excitotoxicity in motor neurons.^51^ Excitotoxicity is a key mechanism linked to ALS^71^ and thus estradiol may be the effector molecule underlying our observation. We carried out a series of orthogonal analyses which support the importance of estradiol. We were able to show in a separate MR that estradiol is itself linked to lower risk of ALS although some caution is necessary because this result relied on the MR Egger estimate in isolation. Our rare variant analysis demonstrated that missense mutations within *HSD17B1* alter risk for ALS although this result only reached nominal statistical significance. *HSD17B1* encodes 17β-HSD1 which is the enzyme responsible for the conversion of estrone-3-sulphate into estradiol. The functional impact of observed missense mutations is unclear but closer examination of the discovered mutations in our dataset revealed two interesting observations: Missense variants at specific sites within 7β-HSD1 have been associated with reduced enzyme activity^72^ but the only one of these sites which was mutated in our dataset was a p.Asp113Tyr change within the dimaeric interface; this change was present in a single ALS patient and absent from controls. Theoretically, this mutation would impair the production of estradiol in this ALS patient. Finally, supplementation of zebrafish embryos carrying an ALS-associated *C9ORF72* mutation with estradiol reduced cellular stress consistent with a neuroprotective effect *in vivo*. Although cellular stress is not specific to ALS, we have previously shown that our readout is correlated with neuronal stress and eventually motor neuron toxicity including loss of neuromuscular junctions.^43,53,73^ Thus, we believe that the modulation of cellular stress by estradiol in our model system indicates a disease-specific effect. We have previously reported a similar result for estradiol in an unbiased drug screen utilizing zebrafish engineered to express an ALS-associated G93R mutation within *SOD1.*^74^

A previous population study provides high-quality evidence that ACE-I is protective against ALS.^46^ The mechanism underpinning this observation is still unclear, but our discovery that bradykinin is protective suggests that the action of ACE-I to inhibit the degradation of bradykinin may be crucial. However, it should be noted that the bradykinin exposure did not remain significant in our replication GWAS, although this may have been a result of the greater population heterogeneity within ALS patients in the replication GWAS.^14,15^ Common variant architecture and LD structure, in particular, varies between populations and this can confound MR analyses.^75^

We have conclusively demonstrated that higher serum isoleucine levels are associated with an increased risk of ALS and this effect is mediated by depletion of vitamin B12. Our MVMR study revealed that the toxic effect of isoleucine was negated if it was conditioned on the protective effect of vitamin B12 or its active form, holotranscobalamin. The link between isoleucine and vitamin B12 is well described clinically.^58^ In an orthogonal step, our rare variant analysis demonstrated that mutations within methylmalonyl-CoA mutase which reduce depletion of vitamin B12 by isoleucine metabolism, are themselves protective against ALS although this result only reached nominal statistical significance. Previously, we discovered that the generation of succinyl-CoA by the TCA cycle is associated with slower disease progression in ALS.^76^ Succinyl-CoA is the end-product of the reaction catalyzed by methylmalonyl-CoA mutase and therefore a higher concentration of succinyl-CoA might be expected to shift the reaction equilibrium point and reduce vitamin B12 depletion.

A link between isoleucine and ALS has previously been postulated: Two clinical trials of BCAA for treatment of ALS noted a significant deleterious effect in patients receiving the active drug which led to premature termination of one of the trials.^6,7^ It has been estimated that the treatment regimen used in this trial increased plasma isoleucine levels between three- and six-fold^77^ so this should not be considered representative of physiological variation with a standard diet. A later epidemiological study attempted to address the relationship between prediagnostic BCAA levels and ALS in a population cohort but found no evidence of a significant relationship.^78^ It should be noted that the only relationship in this study with a risk ratio >1 indicating a possible positive association with the development of ALS, was for serum isoleucine measured less than 5 years before ALS was diagnosed. In addition, this study included only 275 individuals who developed ALS, whereas our MR experiments aggregated data from a minimum of 12 577 ALS patients; it is, therefore, possible that the population cohort study was underpowered.

Our findings have important implications for the field. Currently, we are unable to assess individualized risk of isoleucine-induced ALS, and it is possible that serum isoleucine is not harmful in some individuals, perhaps depending on genetic background. However, BCAA supplements are used commonly by athletes and others as an ‘energy boost’; we would caution against this in individuals with a family history of ALS who might have higher than background risk of developing the disease. Moreover, vitamin B12 is a low-cost and safe intervention that could be administered to at-risk individuals, particularly if they were known to have high serum isoleucine levels. Indeed, testing isoleucine and consideration of vitamin B12 supplementation is potentially an effective form of personalized medicine which could reduce the risk of ALS.

## Supplementary Material

fcac069_Supplementary_Data

## Abbreviations

17β-HSD1 =: 17β-Hydroxysteroid dehydrogenase
ALS =: Amyotrophic lateral sclerosis
ACE-I =: angiotensin-converting enzyme inhibitors
BCAA =: branched-chain amino acid
C9ORF72 =: chromosome 9 open reading frame 72
DMSO =: dimethyl sulphoxide
dpf: days post-fertilization
ExAC =: Exome Aggregation Consortium
GFP =: green fluorescent protein
GWAS =: genome-wide association study
HSR =: heat shock cellular stress response
HSD17B1 =: Hydroxysteroid 17-Beta Dehydrogenase 1
InSIDE =: INstrument Strength Independent of Direct Effect
IVW =: inverse variance weighted
LD =: linkage disequilibrium
LDSC =: linkage disequilibrium score regression
LOF =: loss of function
LMM =: linear mixed model
MAF =: minor allele frequency
MMUT =: methylmalonyl-CoA mutase
MR =: Mendelian randomization
MR-PRESSO =: Mendelian Randomization Pleiotropy RESidual Sum and Outlier
MVMR =: Multivariable Mendelian randomization
PheWAS =: phenotype-wide association study
RCF =: relative centrifugal force
SNPs =: single nucleotide polymorphisms
