# Supplementary material for: Unbiased metabolome screen leads to personalized medicine strategy for amyotrophic lateral sclerosis
Source: Brain Commun. 2022 Mar 17;4(2):fcac069. doi: 10.1093/braincomms/fcac069 (PMC9010771; doi:10.1093/braincomms/fcac069)
Supplement: fcac069_Supplementary_Data [file fcac069_supplementary_data.zip › Supplementary Figure 1.pdf]

Supplementary Figure 1

A

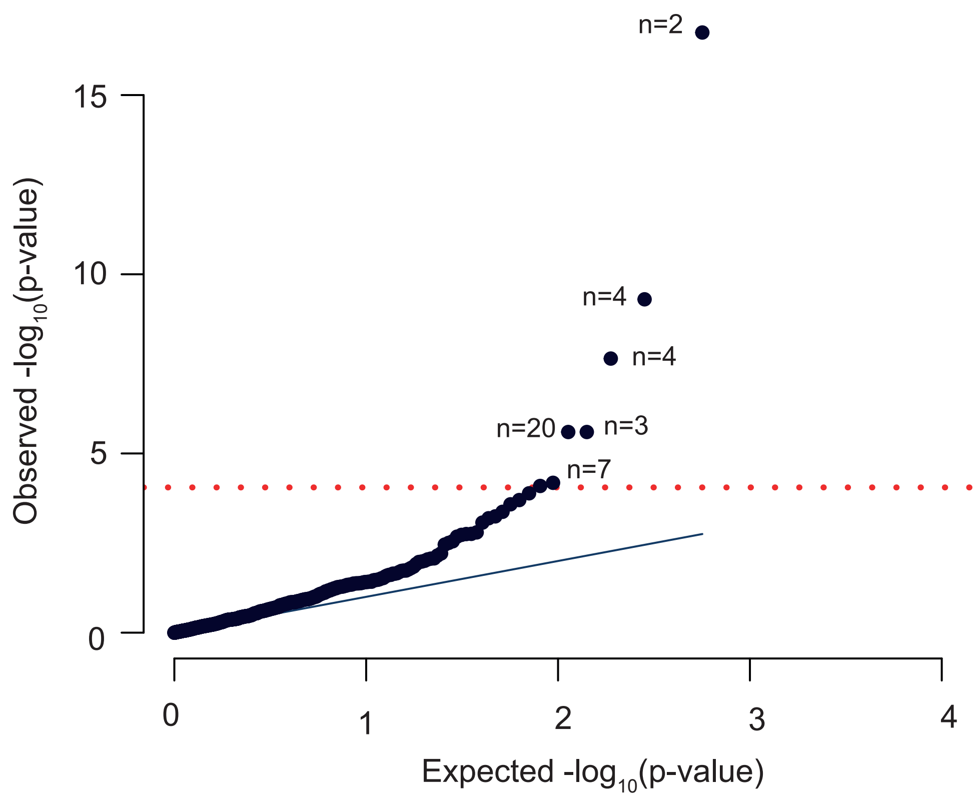

B

Minimim number of instruments	Maximum Number of instruments	λ
No cut off	No cut off	1.38
No cut off	17	1.283
No cut off	16	1.284
No cut off	15	1.23
No cut off	14	1.203
No cut off	13	1.279
3	14	1.23
4	14	1.177
5	14	1.117
6	14	**1.114**
7	14	1.117
8	14	1.23
5	17	1.173
5	16	1.177
5	15	1.118
5	14	1.117
5	13	1.17
6	17	1.233
6	16	1.275
6	15	1.117
6	14	**1.114**
6	13	1.232
