# Supplementary material for: Unbiased metabolome screen leads to personalized medicine strategy for amyotrophic lateral sclerosis
Source: Brain Commun. 2022 Mar 17;4(2):fcac069. doi: 10.1093/braincomms/fcac069 (PMC9010771; doi:10.1093/braincomms/fcac069)
Supplement: fcac069_Supplementary_Data [file fcac069_supplementary_data.zip › Supplementary Figure 2.pdf]

Supplementary Figure 2

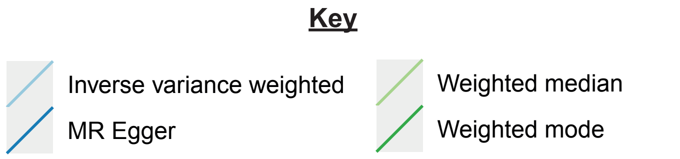

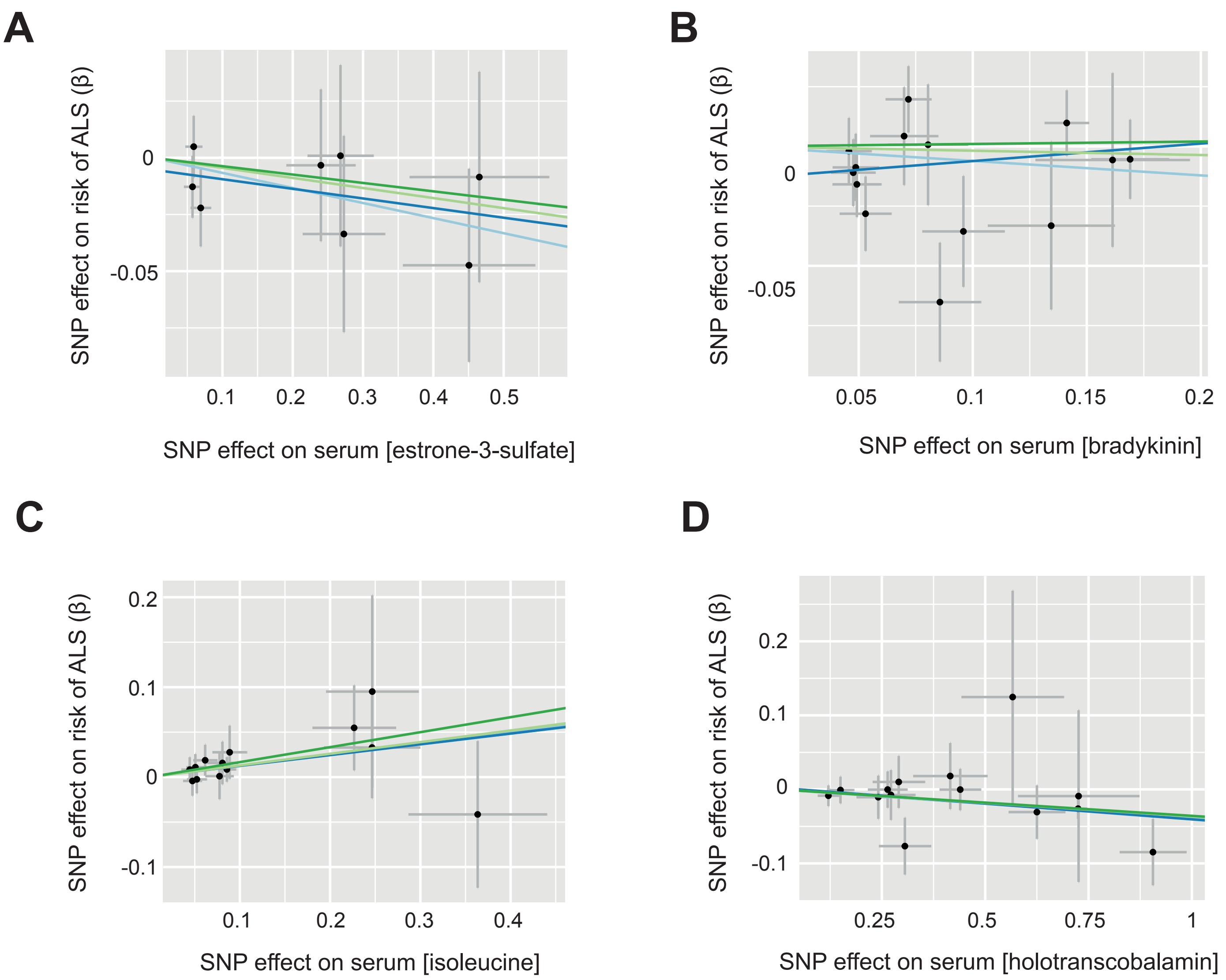

E

Test	Estrone-3-Sulfate	Bradykinin	Isoleucine	Holotranscobalamin
IVW P value	**0.03**	0.3	**0.001**	**0.0004**
Egger P value	0.57	0.57	0.43	0.14
Weighted Median P value	0.5	0.81	0.18	**0.04**
Weighted Mode P value	0.63	0.89	0.24	0.06
MR-Lasso P value	0.18	0.3	0.1	**0.01**
Number of SNPs F<10	0	0	0	0
Egger Cochran's Q test P value	0.86	0.23	0.98	0.87
IVW Cochran's Q test P value	0.9	0.2	0.99	0.91
MR PRESSO	0.91	0.24	0.99	0.92
Egger intercept test	0.64	0.25	0.96	0.87
I2	0.96	0.97	0.97	0.99
Number of SNPs LOO P>0.05	2	14	0	0
Total number of SNPs	8	14	13	14
