# Supplementary material for: Unbiased metabolome screen leads to personalized medicine strategy for amyotrophic lateral sclerosis
Source: Brain Commun. 2022 Mar 17;4(2):fcac069. doi: 10.1093/braincomms/fcac069 (PMC9010771; doi:10.1093/braincomms/fcac069)
Supplement: fcac069_Supplementary_Data [file fcac069_supplementary_data.zip › Supplementary Figure and Table legends_230322.docx]

**Supplementary Material:**

**Supplementary Table 1: IVW MR results for every metabolite tested in an unbiased metabolome screen for ALS risk modifiers.**

**Supplementary Table 2: Metabolites identified in an unbiased screen are significant in multiple robust MR measures.** Robust MR measures and sensitivity analyses for each MR analysis.

**Supplementary Table 3: Metabolites identified in an unbiased screen and their downstream byproducts are significant in a replication ALS GWAS.** Robust MR measures and sensitivity analyses for each MR analysis.

**Supplementary Table 4: MR analysis of additional members of metabolic pathways containing metabolites identified in an unbiased screen.** Robust MR measures and sensitivity analyses for each MR analysis.

**Supplementary Table 5: Rare missense variants identified within *HSD17B1* in 4,366 ALS patients and 1,832 age and sex matched controls.** Rare variants were defined as population MAF<1% [^79^](https://paperpile.com/c/hTNvmI/Vr7w).

**Supplementary Table 6: LOF variants identified within *MMUT* in 4,366 ALS patients and 1,832 age and sex matched controls.** Rare variants were defined as population MAF<1% [^79^](https://paperpile.com/c/hTNvmI/Vr7w). ‘Disruptive’ LOF variants where defined as frame-shift, splice site, exon loss, stop gained, stoploss, startloss, and transcription ablation variants.

**Supplementary Figure 1: Exclusion of MR tests with small and large numbers of instrumental SNPs effectively controls p-value inflation. (A)** QQ-plot demonstrates that there was evidence of p-value inflation in our unbiased metabolome screen (λ = 1.38) before removal of tests with small or large numbers of instrumental variables. Bonferroni multiple testing threshold is indicated by a red line. (**B**) The optimal number of instrumental SNPs was tuned to minimise the inflation factor (λ); ultimately we excluded analyses which used n<6 or n>14 SNPs.

**Supplementary Figure 2: Metabolites identified in an unbiased screen and their downstream byproducts are significant in a replication ALS GWAS.** (**A-D**) Scatter plots demonstrating the correlation between genetic liability to serum estrone-3-sulfate (**A**), bradykinin (**B**), isoleucine (**C**), and holotranscobalamin (**D**), with genetic liability to ALS. Points indicate effect size (β) and standard errors for each SNP-outcome relationship. (**E**) Robust MR measures and sensitivity analyses for each MR analysis.
